# Building on Vaccine Confidence in the Aftermath of the Pandemic: A Qualitative Study in Primary Care Physicians

**DOI:** 10.3390/vaccines14050415

**Published:** 2026-05-04

**Authors:** Ilias Pagkozidis, Georgios Papazisis, Stamatina Driva, Anna-Bettina Haidich, Zoi Tsimtsiou

**Affiliations:** 1 Department of Hygiene, Social-Preventive Medicine and Medical Statistics, School of Medicine, Aristotle University of Thessaloniki, 54124 Thessaloniki, Greece; pagoilias@gmail.com (I.P.); haidich@auth.gr (A.-B.H.); 2 Department of Clinical Pharmacology, School of Medicine, Aristotle University of Thessaloniki, 54124 Thessaloniki, Greece; papazisg@auth.gr; 3 Hippocratio General Hospital of Athens, 11527 Athens, Greece; matina_driva@hotmail.com

**Keywords:** adult vaccination, primary care physicians, primary health care, vaccine confidence, COVID-19, pandemic, qualitative study, Greece

## Abstract

**Objectives:** We aimed to document the impact of the COVID-19 pandemic on the public’s attitudes and stances towards adult vaccination, as perceived by frontline primary care physicians (PCPs), as well as their proposed strategies to boost vaccine confidence and uptake. **Methods:** We conducted semi-structured interviews with 25 PCPs, recruited via purposive and snowball sampling from urban, semi-urban, rural and island regions of Greece. Interviews conducted from January to June 2025 were transcribed verbatim, anonymized and analyzed using thematic analysis. **Results:** PCPs’ views on the impact of the pandemic were conflicting. The pandemic reportedly elevated the importance and necessity of adult immunization, brought immunizations into the patient–PCP agenda, and increased trust in PCPs as well as the uptake of other preventive services. Yet PCPs also underscored the increased difficulty in communicating vaccinations and the spillover hesitancy to vaccines. To strengthen vaccine confidence, PCPs proposed invigorating the public’s health literacy, recommending vaccinations at any PCP–patient encounter, strengthening health workers’ training regarding immunizations and introducing effective health policies on incentives, reinforced PHC services, digital health tools and vaccination sites. **Conclusions:** Despite heterogenous data on the impact of the pandemic on adult immunization, the urgency to address the challenges and seize the post-pandemic opportunities through public health strategies that reflect health workers’ and communities’ needs and values was underscored.

## 1. Introduction

Vaccination is considered among the most effective, efficient and successful interventions in public health [1]. Increasing vaccine coverage among the general population is estimated to avert 1.5 million additional deaths worldwide and ease the economic burden that vaccine-preventable diseases place on health systems [2,3]. To achieve this, health workers’ advice is crucial, as recommendations from trusted advisors are widely reported to increase vaccine confidence and uptake [3,4,5]. Vaccine acceptance and adherence to national immunization guidelines represents a complex behavioral phenomenon that varies according to the type and availability of vaccines, place, time and context of vaccination, as well as individual and community beliefs [6,7,8]. The “5Cs” model aids in understanding the drivers of vaccine acceptance: ‘confidence’ in vaccine safety and effectiveness, health systems and authorities; ‘complacency’ (the perception over one’s risk and susceptibility to disease); ‘constraints’ regarding immunization (availability, accessibility, and affordability of vaccinations); ‘calculation’ of available evidence, information and recommendations; and collective responsibility (the willingness to protect the community) [9,10]. Additionally, circumstantial contact with health workers, missed opportunities during care provision, a lack of care continuity and strong social norms regarding adult immunization contribute to the historically low coverage rates in adults [5,11,12].

Τhe emergence of the SARS-CoV-2 virus highlighted the value of immunization in overcoming the COVID-19 pandemic. Despite the advancements in vaccine research and development, the unprecedented speed of this process amplified hesitancy and doubt among both the general population and health providers. To better reflect the growing hesitancy, the “5Cs” framework was expanded to incorporate key new drivers, such as ‘conspiracy’ thinking and ‘compliance’ with national guidelines regarding immunization and curbing the spread of the novel virus [6]. Factors such as the mistrust in public health authorities, governments and the pharmaceutical industry, religious constructs and beliefs regarding the locus of control over one’s health, along with opposition to compulsory immunizations and missteps in public health communication, further augmented hesitancy [13,14]. Mistrust in immunizations has transcended beyond the novel vaccines, as declining acceptance and an unwillingness to vaccinate against other diseases have been increasingly noted in the literature [15]. Moreover, as health-seeking behaviors shifted and mass vaccination campaigns, health promotion strategies and digital tools were deployed, adults’ approach to preventive services and immunization has been collectively reshaped, potentially influencing the uptake of all recommended adult vaccines.

Gaining insight into the impact on individuals’ behavior regarding vaccinations through the lens of frontline health workers, as well as documenting proposals to strengthen confidence, are imperative in the ever-evolving post-COVID landscape. Currently, the available evidence examining changes in vaccination beliefs and practices of the general population is scarce, focuses on the early stages of the pandemic, and provides limited understanding of the inflicted changes in vaccination behavior in the long run. This study aimed to explore the impact of the pandemic on citizens’ attitudes and practices regarding adult immunization, as perceived by primary care physicians (PCPs), as well as their suggestions on how to strengthen vaccine confidence among the general population, thereby informing the development of public health strategies that reflect community needs, ‘lessons learnt’, and PCPs’ values.

## 2. Materials and Methods

### 2.1. Study Population and Sampling

A qualitative design was employed, utilizing in-depth, semi-structured, individual interviews. PCPs from all regions of Greece were invited to participate. Study participants met the following inclusion criteria: (a) practicing as General Practice/Family Medicine (GP/FM) physicians; and (b) working in Primary Health Care (PHC) units, public or private, at the time of the study. To strengthen outreach and facilitate recruitment, an invitation was sent to members of a GP/FM professional association. Purposive and snowball sampling methods were utilized, accounting for participants’ gender, work experience, area of work and the public or private status of their practice. Additionally, participants were asked to share the study invitation with their professional network to maximize outreach. Participants had no prior established relationship with the research team. No incentives were provided, and participants were informed of their right to withdraw from the study and have their data removed at any given time without repercussions. Participant recruitment was conducted concurrently with data analysis and ceased once saturation was achieved. No follow-up interviews were required.

### 2.2. Data Collection

An interview guide, developed based on an extensive literature review and expert consultation, was pilot-tested with three PCPs and subsequently reviewed by the research team. The latter further refined the interview guide by incorporating additional feedback from a researcher experienced in qualitative study design. The final interview guide aimed to explore PCPs’ stances, knowledge, practices and proposals to strengthen vaccine confidence. This article reports on the qualitative insights of PCPs regarding the effects of the pandemic on adult vaccination practices, and their suggestions for increasing vaccine confidence (File S1). The interviews were conducted remotely via teleconferencing platforms (Zoom and Microsoft Teams) between January and June 2025. They were conducted outside of office hours by a physician experienced in qualitative methodology. All interviews were audio-recorded with prior consent, transcribed verbatim and any identifiable data was anonymized. The transcripts were checked for inconsistencies by comparing them against field notes taken during the interviews.

### 2.3. Data Analysis

To analyze the interview data, thematic analysis was conducted [16]. Data from the first three interviews, which were used to pilot test the interview guide, were excluded from the analysis. Transcription and data processing were executed independently by two researchers (IP and SD), who are PCPs themselves with experience in conducting qualitative research. An inductive approach was utilized to identify and formulate concepts and themes from raw data [17]. Guided by the Braun and Clarke framework [16], the researchers first familiarized themselves with the initial interview transcripts and drew preliminary codes. They subsequently began open coding the available transcripts, collecting data and relevant context for each code. Codes were grouped into preliminary sub-themes and overarching themes, which were then reviewed to assess their fit with the respective extracts and the overall transcripts. The researcher team then met to compare their identified themes, sub-themes and individual codes. Differences in coding and divergent interpretations were resolved through group discussion until consensus was reached. A third, independent researcher (ZT) then verified the analysis. A common coding framework was developed, agreed upon, applied and iteratively refined across all available transcripts. A sample coding tree illustrating the theme development process is provided in File S2. Data collection and processing occurred concurrently to monitor for data saturation. Adhering to Hennik et al.’s framework [18], we distinguished and evaluated both coding and meaning saturation. Coding saturation was considered reached once no new codes were identified in three consecutive transcripts. Meaning saturation was considered reached once no new dimensions or insights regarding the existing codes emerged, indicating a comprehensive understanding of the studied phenomenon. Ultimately, we adhered to the Standards for Reporting Qualitative Research (SRQR) during the design, implementation and reporting of this study (File S3) [19].

To minimize personal bias in study design, as well as during collecting, analysis and reporting of the data, a systematic bracketing approach was followed. All researchers are vaccine advocates, keen to increase vaccine confidence within their community and among colleagues. During the study design, researchers discussed their attitudes regarding the issue, acknowledged their personal values and biases, and developed the interview guide. The latter was then reviewed by an independent researcher outside the healthcare profession, whose feedback aided in rephrasing the guide to avoid eliciting socially desirable responses. During the interviews, the interviewer remained continuously aware of their own stances, maintained an impartial demeanor, and sought to explore opposing perspectives. The recruitment process was considered to minimize social desirability bias, as the lack of any a priori acquaintance with the interviewer encouraged truthful responses. Throughout the analysis, reflexive discussions were conducted among the researchers coding the data. Emphasis was placed on coding and interpreting the evident content of the data. To guard against potential under-coding or de-emphasizing opposite perspectives, peer debriefing was utilized. This guided the exploration, interpretation and inclusion of opposing views, ensuring truthful representation in the final analysis, as they emerged from raw data.

### 2.4. Ethics Approval

This study was conducted in accordance with the Declaration of Helsinki and approved by the Bioethics Committee of the School of Medicine, Aristotle University of Thessaloniki, Greece (Decision No 247/2024-2/7/2024). All participants signed informed consent forms at recruitment.

## 3. Results

In total, 25 PCPs from 11 prefectures (spanning both mainland Greece and the islands) took part in the interviews. The study participants were predominantly female (60%). Regarding geographic distribution, 11 PCPs practiced in urban areas and 9 in rural areas, with the vast majority (92%) practicing in public PHC units. The PCPs’ characteristics are presented in Table 1. The mean duration of the interviews was 47.8 min (±13.7, min. 29–max. 92).

### 3.1. The Impact of the Pandemic on Public Attitudes and Practices Regarding Adult Vaccination

Participants’ views regarding the perceived effects of the pandemic on attitudes and practices toward adult vaccination were conflicting. While the majority of Greek PCPs stated that vaccine confidence was weakened in the aftermath of the pandemic, several participants argued that vaccine confidence and acceptance were bolstered in the post-pandemic landscape. An inability to recognize potential changes was highlighted by two participants due to the lack of prior engagement with routine immunization discussions pre-pandemic, while three participants underscored an absence of change in the general population’s beliefs on the issue. The three different standpoints are outlined below.

#### 3.1.1. Strengthened Vaccine Confidence and Increased Utilization of Preventive Services

In the aftermath of the pandemic, the already close and unique trust-based relationships between PCPs and their communities were strengthened. Health workers became increasingly aware of the importance of vaccination, as well as the evident benefits of boosting uptake and coverage. Immunization was thus placed at the forefront of the patient–physician agenda, with participants noting that hesitancy toward the novel vaccines did not transcend to more established ones. Although vaccine fatigue may have initially led to delays in routine vaccinations, this phenomenon has receded post-pandemic. A deeper understanding of the value of immunization facilitated discussions even with hesitant patients and yielded positive outcomes, while the surge in vaccine research and development led to the increased recommendation and uptake of vaccinations. Furthermore, the tangible impact of PCPs’ recommendations to immunize against SARS-CoV-2 reportedly accounted for citizens’ broader adherence to other preventive services proposed by their physicians. The main themes that emerged regarding the positive impact of the pandemic on vaccine confidence and utilization of preventive services, along with illustrative quotes, are displayed in Table 2.

#### 3.1.2. Weakened Vaccine Confidence

Most participants argued that citizens’ practices and confidence in immunization dwindled following the pandemic, manifesting as: (a) increased effort and difficulty in communicating, discussing, recommending and persuading individuals over vaccinations; and (b) a spillover of SARS-CoV-2 vaccine hesitancy toward established adult (pneumococcal, seasonal influenza), newly released (e.g., herpes zoster, RSV), and even childhood immunizations. The inundation of adverse messages, negative connotations regarding immunizations, a lack of trust in medical professionals, and the deterioration of patient-provider relationships augmented these challenges. PCPs reflected on the key drivers of this change, which are summarized into eight main themes and are presented alongside illustrative quotes in Table 3.

*People’s trust in vaccines is on a downward spiral… things were not like this before… they have become much more difficult now… COVID-19 damaged trust and people are not easily convinced about immunization. P4*

*Perhaps the COVID-19 vaccine dragged down some new vaccines as well, like RSV, herpes zoster… There was some hesitancy toward the novel vaccines that appeared on the market… Substantial hesitation over the RSV vaccine was not present, it was just the whole fatigue with respiratory viruses. P17*

*I think there is greater hesitation toward other vaccines as well, even the children’s ones. I think a high degree of hesitation exists in parents… Instead of raising people’s awareness about vaccines, I consider the pandemic to have caused long-term damage. P5*

#### 3.1.3. No Remaining Change in Vaccine Confidence

Few PCPs perceived that the pandemic had no effect on the general population’s beliefs and practices regarding immunization. Confidence in the established vaccines was reportedly unaffected, and the initially noted vaccine fatigue was ultimately overturned.

*I haven’t noticed anything extraordinary regarding vaccination. Maybe during the first 2–3 years, citizens were fed up and did not want to vaccinate again. But in the past two years things have changed. P22*

*The established vaccines were not affected. There was no doubt, no one asked us ‘why should we vaccinate against pneumococcus?’… people come with the same frequency and ask for them. P23*

### 3.2. Key Strategies to Strengthen Confidence in Adult Vaccinations

Based on their experience, participants suggested the strategies they considered important for boosting the general population’s confidence in and uptake of adult vaccinations. The main themes that emerged were: a. Strengthening the general population’s health literacy; b. Recommending vaccination at every primary care encounter; c. Reinforcing health workers’ relevant training; and d. Implementing health policies that promote immunization. Each main theme and its corresponding subthemes, where applicable, are presented below alongside illustrative quotes.

#### 3.2.1. Strengthening the General Population’s Health Literacy

All PCPs underlined the need to increase health literacy regarding immunization among the general population through: (a) mass awareness campaigns; (b) community-based actions; and (c) school interventions.

*Awareness campaigns*. Mass educational campaigns were considered of immense value. In stark contrast to practice-level initiatives, which solely target individuals visiting PHC units, organizing awareness campaigns reportedly yields greater benefits. They encourage a broader audience to feel confident and nudge them to discuss vaccination with their PCP. However, to ensure high impact, PCPs stressed the importance of allowing time to recover from conflicting, inundating messages and pandemic fatigue.

*The more aware citizens are, the easier it is to convince them of something therapeutical or to change something as part of health promotion… I wish everyone was informed, as then they would come and ask us [for vaccines]. P15*

Regarding the stakeholders designing and running communication campaigns, our PCPs agreed on the key role of national authorities, such as the National Health System, the Regional Health Authorities, the National Public Health Organization and the Ministry of Health. Viewed favorably by the general population, they represent the ideal actors to organize and deliver awareness messages that: (a) align with national strategies; (b) prioritize vaccinations according to local epidemiological data; and (c) integrate expertise from specialized personnel, such as psychologists, to increase outreach and effectiveness. Professional associations, as well as academic institutions, were considered essential in supporting the efforts of national actors. Though pharmaceutical companies’ awareness efforts were lauded, our PCPs proposed limiting the role of private entities. Privately run campaigns were reportedly unable to advance vaccine confidence in the long run, due to (a) negative public perceptions and connotations of the pharmaceutical industry; and (b) a misalignment between their priorities and those of the national immunization strategy. However, one PCP did propose the introduction of public–private partnerships to achieve greatest outreach and potential.

*An awareness campaign from official national actors… they are more valid institutions… the Health Ministry, the National Public Health Organization and academics can produce campaigns that revolve around information about the disease, the importance of vaccination, [and] how one can get vaccinated. P9*

According to our PCPs, the content of the proposed campaigns should primarily focus on (a) the value, benefits and potential adverse effects of a given vaccine; (b) the research and phases of vaccine development; (c) the official recommendations; and (d) the process of vaccination. Participants noted that in all cases, vaccine misinformation and citizens’ concerns should be directly addressed, and scare tactics must be avoided. Our PCPs further underscored the need to utilize social media, as well as artificial intelligence tools, to effectively deploy vaccination campaigns and strengthen community education. Alongside these approaches, traditional means such as leaflets and TV/radio campaigns were proposed to maximize outreach.

*I think that you should target distrust and showcase one to two examples of how vaccines are developed and that this is not a simple process. [You have to communicate] that the worries one might have about the effects of the vaccine are studied along with other factors. P8*

Regarding the campaign’s ambassador, divergent standpoints were highlighted, with PCPs split between health workers and public figures. High-profile, widely accepted celebrities acting as messengers may increase outreach, attract attention and sway the community towards accepting vaccinations, as past experiences have suggested. Conversely, health workers act as trusted messengers who can instill confidence in vaccines, provided that the public is not overwhelmed by their academic achievements and constant media presence. Moreover, if health workers are invited to headline an awareness campaign, PCPs noted they should avoid overexposure and strictly reflect the national immunization strategies. PCPs further noted the inclusion of patients in such initiatives and sharing of their lived experiences and personal struggles with the disease to foster trust and identification among the public.

*Utilizing personal stories, viewing how people experienced the disease may increase the public’s awareness. Patients who have contracted the disease should be put forward, highlight the issue and convince people to vaccinate. P11*

*Community-based interventions*. Grassroots health interventions were also deemed vital for boosting health literacy. Honest, open discussions with the community were deemed highly effective in increasing vaccine confidence and uptake, especially among vulnerable groups. Organizing such actions reportedly requires a needs assessment survey by local units, thoughtful planning of the delivery (in terms of location, time and target population) and an evaluation of the intervention and its proposed outcomes. To maximize participation and outreach, synergies with other stakeholders—such as municipal authorities, schools and the church—should be sought.

*Within the scope of public health, you can do a group briefing. You can utilize the acceptance of and familiarity within the group to help convince others to vaccinate. You can solve queries that may arise. If you respond to their doubts, you build a wall against distrust. The more our influence grows, social acceptance of vaccines changes… and this creates synergies, as people see how their counterparts react… P8*

*School interventions*. Study participants underscored the significance of increasing awareness and promoting vaccination at an early age. Bottom-up approaches, such as school-based initiatives, would not only strengthen trust and confidence among future adults but also aid in informing their parents about the importance of immunization at all life stages.

*Individuals are ‘formed’ in elementary schools. This is where the job must be done. We should remind children that vaccines have saved us. Like all campaigns, the vaccination one should start in elementary school. What one learns at a young age is what sticks forever. P14*

#### 3.2.2. Recommending Vaccination at Every Primary Care Encounter

Discussing recommended vaccinations according to national guidelines and advising patients toward immunization constituted an integral part of the patient–physician encounter. Considering the allocated time and clinical context, PCPs emphasized that immunization should be brought up during every patient–provider contact, ranging from routine visits to consultations in the emergency department. PCPs, as trusted providers, are uniquely equipped to educate and urge their patient population to vaccinate.

*Every routine visit constitutes a good opportunity. They come to [get] medicine prescribed or do a check-up they think they need, and it’s a great opportunity to put everything in order, including vaccinations. In the emergency department, it depends on the workload. P3*

#### 3.2.3. Reinforcing Health Workers’ Relevant Training

All PCPs underscored the significance of educating health workers on immunization. Building a pool of informed health workers is crucial for addressing their own hesitancy and concerns, thus allowing them to accurately educate the public and follow best practices. Soft skills training empowers providers to communicate and recommend vaccinations with self-efficacy and effectiveness, confidently recognize individuals’ stance on the continuum of vaccine acceptance, and guide discussions accordingly. National stakeholders, professional associations, and universities were perceived to be the primary entities involved in training PCPs. However, study participants also noted the importance of pharmaceutical representatives and non-formal learning modalities, such as the use of tables, posters, and other visual cues on their PHC practice.

*Seminars that include skills to approach patients, with relevant examples and clinical scenarios with different types of patients. It is also a matter of communicating things… the scientific knowledge exists and you may acquire it, but if you cannot communicate it, understand the individual across [from] you, and win their confidence, it’s difficult [to convince them] … this short intervention technique is difficult and needs practice. Communication skills regarding vaccinations were part of a whole seminar in my case. P4*

#### 3.2.4. Implementing Health Policies Promoting Immunization

PCPs underscored the significance of advancing immunization through strategic health policies, including (a) establishing incentives; (b) strengthening PHC services; (c) establishing an adult vaccination registry; (d) utilizing digital health tools; and (e) offering vaccinations at PHC units and pharmacies.

*Establishment of incentives*. In an effort to increase engagement with the issue, PCPs proposed establishing incentives for both citizens and health workers.

Though providing discounts at pharmacies and for health services was deemed positive, our participants stood firmly against the establishment of direct financial incentives toward the general population. Financial rewards reportedly weaken the public’s confidence, diminish the value of adult immunization, and can have a ripple effect on the adoption of childhood vaccinations. Perceived as a kind of transaction, they were viewed unfavorably, as they reportedly validate conspiracy theories and were not deemed feasible within the broader cultural context.

*I don’t think this would work. It’s like a boomerang… a kind of commercialization… paying you to get vaccinated? It won’t work. Greeks are born suspicious, so paying them to do something is surely not the way to make them trust it. P5*

Conversely, financial incentives for health workers were positively regarded. Following the precedent of other preventive medicine campaigns, remuneration would reportedly motivate PCPs, practically acknowledging and propelling their efforts to safeguard public health. Yet, PCPs cautioned that remuneration ought not to be a PCPs’ sole primary motivation, as health promotion and immunizations are considered an integral part of GP/FM. To minimize potential backlash, PCPs suggested increasing their total remuneration scheme to incorporate but not explicitly publicize vaccination-specific benefits.

*It is an integral part of our discipline, and we should not rely solely on incentives to vaccinate individuals. No one would like this and the public would argue that since we are getting paid to prescribe them, they are being experimented upon. P16*

*Drawing from experience, it was evident that physicians that did not care for their patients that much, suddenly became aware and were striving to persuade individuals to undergo screening tests. This means something. If they had a financial motivation, most PCPs would care more about informing their communities. P23*

*Strengthening PHC*. Building upon the longstanding partnerships established with citizens throughout their healthcare journey, expanding and invigorating the current scheme of PCPs is crucial for boosting vaccine confidence. Elongated patient encounters allow PCPs adequate time to discuss and recommend vaccinations. Our participants also called for the establishment of specialized adult vaccination health teams, with increased participation from nurses and health visitors who are already familiar with childhood immunizations. Fostering a welcoming culture toward vaccinations within a PHC unit and aligning health workers’ interactions with the public toward a common goal were considered priorities.

*What is the PHC unit’s culture? What do they convey regarding immunizations? Do we have a united ‘front’? If we have an honest discussion, training and culture building, we can make it happen. It can start with us, the health providers. The PHC unit must be a united front. Culture building, appropriate behavior and having the whole unit support such initiatives [are key]. P25*

*Vaccination registry*. The creation of an adult vaccine registry is key to tracking real-time coverage and uptake, as well as enabling the collection of quality metrics. Diverting from the currently fragmented and unreliable system, which solely records prescribed vaccinations, and building upon the framework of the COVID-19 vaccination registry, PCPs noted that only performed immunizations should be recorded in the adult database. Following the footsteps of the childhood registry, diverse health disciplines should be able to access and input data.

*There should be a record… we should be organized… we should know, beyond any doubt, what has been done, when and by whom. Right now, we are counting on everyone’s memory and honesty. We have to believe whatever they say. P5*

*Digital health tools*. Capitalizing on the success of the current e-health app, PCPs proposed the introduction of direct, personalized reminders via the app, email or SMS that prompt individuals to vaccinate and contact their PCP. To spark discussions with the latter, participants proposed launching a dedicated website or providing a specific section within existing health apps that addresses frequently asked questions and offers information about the immunization procedure.

*The best way to spread awareness and the official recommendations is through a text message, for example. A text message on Viber indicating that citizens belong in this group, should get vaccinated, and should talk with their physician. P3*

*Vaccination sites*. Neighborhood pharmacies and PHC units were considered optimal vaccination sites. The former constituted a familiar environment for individuals, reportedly enhanced the perceived safety of immunization, boosted confidence and offered pharmacists the opportunity to counsel and refer individuals to PHC units. Point-of-purchase vaccination minimized the likelihood of patient dropout and missed vaccinations. As long as pharmacists are prepared and trained to manage complications, PCPs supported expanding their role in vaccination campaigns beyond seasonal influenza. On the contrary, vaccinating within PHC units reportedly increased compliance and confidence, as health workers could simultaneously consult, recommend and vaccinate patients, thereby minimizing potential mistakes in dispensaries. Ultimately, prescribing and performing vaccinations on specific days, or offering mass vaccination events, would reportedly ease the everyday workload in PHC units and prove vital for enhancing uptake in remote areas.

*It is simpler to get vaccinated in a pharmacy. The shot gets demystified and people are not scared. The level of trust [in vaccines] is elevated as the health provider in your neighborhood, the one who takes good care of you, is the one performing the vaccination. P5*

## 4. Discussion

### 4.1. Main Findings

Building upon the existing literature regarding post-pandemic vaccine confidence, we investigated this complex phenomenon at a unique intersection by focusing on adult vaccination in the Greek healthcare context, as perceived by frontline PCPs. Moreover, this study maps PCPs’ proposed strategies to enhance vaccine confidence and uptake among adults. Heterogeneity of perspectives was revealed in the findings, with participants noting that the pandemic highlighted the importance of and need for adult vaccinations, placed related discussions on the patient–physician agenda, and increased trust in PCPs and the uptake of other preventive services. Yet, the post-pandemic era was also reportedly characterized by increased effort and difficulty in discussing vaccinations, as well as a spillover of vaccine hesitancy. To boost vaccine confidence and coverage, PCPs proposed strengthening the public’s health literacy though awareness campaigns, community and school-based interventions, alongside recommending immunization at every patient–physician encounter. The need to bolster relevant training for health workers was underscored. Ultimately, PCPs deemed it imperative to implement effective health policies, including the establishment of economic incentives and vaccine registries, strengthening PHC services, utilizing digital tools, and expanding vaccination sites.

### 4.2. Changes in Attitudes and Adult Vaccination Practices After the COVID-19 Pandemic

In line with our findings, heterogeneity regarding the effect of the pandemic on vaccine attitudes and practices is noted in the literature. Inconclusive evidence has been reported in other studies as well, with half of Polish individuals considering their stances to have remained unaffected and only vaccine advocates reporting increased trust [20]. Siani and Tranter communicate that the UK public’s attitudes toward vaccine confidence remained largely unaffected based on self-reported data [21]. Likewise, the health crisis did not impact the decision to vaccinate against seasonal influenza in a study conducted among caregivers and contacts of vulnerable children in Greece [22]. Conversely, reflecting our participants’ thoughts on the upsurge in preventive behaviors, Vojtek et al.’s review underscored a favorable shift in stances regarding immunization, highlighting the health crisis as a primary driver of vaccine uptake for seasonal influenza and pneumococcus [23]. Complex concepts such as vaccines, immunology, antibodies and viruses were undeniably spotlighted during the pandemic [24]. Continuous exposure to and the popularization of these concepts have led to increased awareness among the general population and they thus presently constitute an integral part of the patient–provider consultation agenda. Although trust in healthcare and health workers was strong at the onset of the crisis, it has receded post-pandemic [25,26].

In the post-pandemic era, our PCPs observed deteriorating stances and a need for increased effort to persuade individuals to undergo adult vaccination. Safety concerns regarding the COVID-19 vaccines and a low risk perception hindered confidence [27,28,29,30]. Although compulsory vaccination measures may temporarily enhance uptake, their adoption ultimately dwindles confidence and increases vaccine refusal [30,31]. Ortiz-Prado et al. argue that the perception of the novel vaccines as life-saving was eventually reversed, undermining their perceived importance [27]. The unprecedented media exposure and attention directed toward the disease and the novel vaccines, combined with the intense and severe disruptions to individuals’ lives, allowed SARS-CoV-2 vaccine hesitancy to drive hesitancy toward other vaccines [15]. Despite increased uptake during the crisis, a survey among US nationals underscored reduced compliance with seasonal influenza vaccination post-crisis [32]. Similarly, a UK study underpinned a significant reduction in vaccine confidence, measured via both self-reporting and validated tools [21]. Echoing our PCPs’ concerns, a sharp reduction during the health crisis followed by slow recovery rates has also been reported for childhood immunizations across the globe [23,27,33].

As the pandemic was closely monitored by the mass media, public health communication strategies were constantly evolving and adapting to current epidemiological conditions. These necessary shifts in narrative fueled vaccine hesitancy and distrust in institutions and their strategies to mitigate the pandemic, as contradictory messaging was often viewed as proof of incompetence [24,27,34]. An abundance of misleading claims and hoaxes further hindered citizens’ efforts to locate credible sources of information [24,27,30]. Perceived as trusted messengers, the public presence of health professionals aided in understanding and communicating the threat and protective measures, as well as evoking compliance with public health responses [3,4,5,20]. Yet, repetitive and excessive exposure to the same messengers ultimately contributed to fatigue and increased resistance [35].

Vaccine acceptance is, ultimately, a phenomenon influenced not solely by evidence-based information and policies, but also by broader cultural contexts and psychosociological determinants [6,7,8]. As such, individuals hesitant toward immunization in the pre-pandemic period may have validated their own beliefs and bolstered their resistance, even when the efficacy of the novel vaccines was evident and despite the severity of COVID-19 illness [36]. A study among Polish citizens revealed that vaccine challengers were more likely to report increased resistance in the aftermath of the pandemic [20]. Mirroring our findings, unmet expectations and related framing regarding the effectiveness of vaccines in curbing the spread of the virus and preventing infection fueled hesitancy among Irish citizens [30].

### 4.3. Strategies to Strengthen Confidence in Adult Vaccinations

Invigorating the public’s health literacy though awareness campaigns was considered of the highest priority in efforts to increase vaccine confidence. In accordance with our findings, international and national public health institutions consistently rank among the most credible stakeholders in public health communication and are key in designing and delivering awareness programs [30,34]. Esteemed within their communities, health workers also constitute key actors in public health messaging [34,37]. Regarding campaign content, tailoring messages according to the recipient’s age is deemed beneficial; research highlights that themes of social altruism and personal health risk perception appeal to younger and older age groups, respectively [38]. Proactively debunking misinformation is considered more effective than reactive approaches to beliefs that might have already become consolidated within the general population [27]. Reflecting our participants’ views, Okuhara and Kiuchi underscored the importance of highlighting patient experiences in public health communication, as personal narratives capture the audience’s attention and foster identification with the messenger [35]. To maximize impact, efforts to strengthen health literacy should move beyond the sole transmission of facts. New frameworks for effective health communication pursue dialog and active participation of society in the production and distribution of accurate, straightforward and culturally sensitive information [24,27]. Finally, utilizing online spaces and digital means of communication is integral to public health communication in the post-COVID era [30,39].

Community and school-based interventions were also considered key in promoting vaccine confidence. Engaging with communities, gaining insight into their perspectives and utilizing these findings to optimize vaccination rollouts are considered essential [40]. PCPs, as the most preferred sources of information even among hesitant individuals [41], are uniquely equipped to lead efforts within the community. Organizing practice-scale interventions that promote patient–provider dialog increases trust and acceptance of vaccines, reportedly boosting vaccination rates by 80% [42]. Forming social synergies with other trusted figures, such as religious leaders and local authorities, is vital to maximize outreach and participation in grassroots-level actions [27]. When such interventions are not feasible, the provision of leaflets and visual cues, such as posters in clinics, serves as a valuable reminder for health providers and the public alike [43,44]. Delivering interventions in schools has proven valuable in raising awareness among students, whilst having a positive spillover effect on parental attitudes toward vaccines [45].

Training health workers on both vaccination and soft skills is integral to instill trust and confidence in vaccines [40]. The provision of recurrent, high-standard education at the undergraduate and lifelong learning levels combats doubts and hesitancy among health workers themselves [31]. Health workers who are deeply aware of the value, safety and efficacy of vaccinations are more likely to recommend and discuss them with their patients [37]. Highly equipped PCPs evaluate patients’ immunization status at every encounter, avoid missed opportunities in care, and tailor their approach according to the latter’s standpoint in the continuum of vaccine acceptance [46]. Their clinical approach and recommendations can significantly increase intention to vaccinate [47], as they constitute strong influencers of immunization decisions for their patients [31].

The establishment of an electronic registry for adult immunizations provides insight into the status of every individual, facilitating the tracking of real-time coverage and supporting pay-for-performance schemes. As also perceived by our PCPs, the establishment of digital reminder systems has proven highly effective in the literature [42,43,44,45]. Liu et al. demonstrated that recall systems targeting patients can increase vaccination by nearly 40%, while those targeting health providers can boost rates by up to 75% [42]. In a similar manner, Ceccarelli et al. underscored that automated reminders via e-health portals or personalized invitations issued by PHC practices and PCPs aided in strengthening vaccine coverage within the community [43].

The provision of financial incentives has been widely tested in international studies. Although perceived as inefficient for strengthening the public’s confidence within the Greek context, providing monetary rewards to patients has been proven to increase uptake in both developed and low-to-middle income countries [42,45,48]. Conversely, providing remuneration to PCPs for strengthening their patient population’s coverage has indeed proven effective in increasing uptake [42,48] and was considered an acceptable strategy by our study participants.

Regarding vaccination settings, our PCPs noted that immunizations should be performed in PHC units, in pharmacies, and during mass community actions. Fisher et al. also identified physicians’ practices as the preferred location for performing immunizations, followed by local dispensaries [41]. Pharmacy settings—which may be the only accessible PHC sites in rural and remote areas—normalize a given vaccine, minimize associated anxiety, promote health literacy in neighborhood settings and instill trust in this ‘routine’ procedure [49]. Furthermore, vaccinating in familiar environments, such as pharmacies or even places of worship, has been proven to increase uptake within communities [45].

Capitalizing on the increased post-pandemic trust in PHC services, the awareness of the value of immunization, and the renewed interest in primary and secondary prevention schemes is key to design effective policy proposals and practice updates that strengthen vaccine confidence and uptake. Within the context and constraints of the Greek PHC system, drawing from the experience of mass screening programs and utilizing existing health apps to provide notifications for pending and available vaccinations are expected to ignite interest and prompt discussions with PCPs. To successfully implement a notification scheme and to adhere to international guidelines on the importance of documenting performed immunizations [46], the establishment of an adult vaccination registry is imperative. Bolstering training on vaccines and communication strategies empowers PCPs to consistently recommend and effectively persuade individuals to vaccinate. Ultimately, facilitating the delivery of vaccines in neighborhood settings and empowering pharmacists to prescribe and administer eligible vaccines beyond seasonal influenza will strengthen trust and increase uptake among the general population.

### 4.4. Limitations of the Study

Certain limitations may affect the interpretation of our findings. First, some views and perspectives may not have been captured. Purposive sampling through a professional association and subsequent snowball sampling may have introduced selection bias, as the resulting sample may be homogenous in terms of practice, stances and interest in vaccinations. Yet, purposive and snowball sampling allowed the research team to reach out and recruit PCPs of diverse age and work experience in PHC from urban, semi-urban and rural areas, across both mainland and island regions. Additionally, the underrepresentation of private practice PCPs in our study may limit the generalizability of our findings to private settings. Yet, this distribution reflects the structural and contextual reality of the Greek PHC system, which is predominately delivered via public PHC units and providers. Furthermore, as physicians discussed their own practices, social desirability bias may have positively influenced their responses to align with perceived ideals of professional conduct. Nonetheless, we consider this bias to have been minimized due to the lack of prior acquaintance with the interviewer and the strict anonymization of transcripts and results. Utilizing teleconferencing platforms for individual interviews may have discouraged PCPs who are unfamiliar with such tools from joining the study. Yet, conducting interviews remotely provided the opportunity to enroll participants from diverse geographical regions and backgrounds, allowing them to recount their experiences in familiar environments such as their homes. Moreover, this study reports on the effect of the pandemic on citizens’ behavior, as perceived by frontline PCPs, alongside their proposed strategies to increase confidence and uptake. As such, only one side of the patient–provider encounter is captured; the findings may therefore not fully reflect the public’s own beliefs or the overall efficacy of PCPs’ proposals.

Because our findings are embedded within the context and organizational constraints of the Greek PHC setting, they may have limited generalizability to countries with different resources and PHC environments. However, as the findings align with the broader international literature, we consider our participants’ perceptions, concerns, and proposals to reflect those of their international counterparts. Ultimately, while our qualitative approach allowed for an in-depth analysis of PCPs’ perspectives, the sizes of our demographic subcategories were inherently small. Therefore, one cannot reliably analyze or argue that observed differences in findings and conflicting views are correlated with specific demographic variables. To build upon and further contextualize PCPs’ stances, beliefs, perceived impact, and the feasibility and fitness of their proposed strategies, appropriately designed cross-sectional studies are to be conducted.

## 5. Conclusions

This study’s findings regarding the impact of the COVID-19 pandemic on the public’s stances and practices toward adult immunization, as perceived by frontline PCPs, were heterogenous. Intensified efforts to discuss, recommend and perform vaccinations are reportedly the norm, with SARS-CoV-2 vaccine hesitancy fueling resistance to longstanding, ‘traditional’ and even childhood immunizations. Yet, the tangible impact and value of COVID-19 vaccinations in overcoming the pandemic have positively influenced the general population, increasing both trust in PCPs and the adoption of vaccines and other preventive measures. Despite the diversity of PCPs’ views, a clear and urgent need to seize opportunities, address the challenges, and take strategic action was underlined. PCPs underscored the urgency of strengthening health literacy at the population, community and school level; the need to train health workers on immunization; and the importance of recommending vaccination at every appointment. Establishing an adult vaccination registry, introducing incentives for PCPs, strengthening PHC services, and allowing for vaccinations in familiar healthcare settings were considered key to combating hesitancy.

## Figures and Tables

**Table 1:** Sociodemographic and professional characteristics of participants (n = 25).

Characteristic	n (%)/Mean (SD, Min.–Max.)
Age (years)	44.9 (8.3, 32–62)
Gender	
Female	15 (60%)
Male	10 (40%)
Number of participants by PHC settings’ location	
Urban	11 (44%)
Semi-urban	5 (20%)
Rural	9 (36%)
Status of PHC unit	
Public	23 (92%)
Private	2 (8%)
Years of professional experience	10.2 (8, 1–26)

**Table 2:** Strengthened vaccine confidence and increased utilization of preventive services after the COVID-19 pandemic described by main themes and illustrative quotes.

Main Theme	Quote
Strengthened trust in PCPs	*In PHC, where our relationship with patients is inherently close, this experience further reinforced that bond, as [the vaccine] we recommended had a tangible impact on their lives and society. P6* *The need for, the trust in, and the relationship with PHC was strengthened. P8*
Recognition of the value of immunization	*We managed to win a significant challenge in PHC in terms of the public’s trust in vaccines. The vaccine was proven to be effective, safe and able to pull us through the health crisis. P6* *The value of immunization was brought to the forefront. In the long run, this can be beneficial, as vaccination, which was irrelevant until now for most citizens, is now a question. When one manages to put a question in someone’s mind and elicits a positive response, this could lead to greater outcomes for the population. P8* *Because the scientific society has turned to prevention and recognized that vaccination constitutes the primary mode of prevention. It’s a weapon at our disposal. The pandemic brought vaccination to the forefront. P10* *The memories are still fresh and thus the public easily recognizes the value of immunization. It allowed them to live and return to normal with safety. P19*
Highlighting the need for adult vaccination	*Never had society and health workers alike been so aware… what we have witnessed in the past 5 years post-COVID was nonexistent before. Adult vaccination was not a matter of much discussion. P9* *It is now more likely for patients to initiate the conversation and ask about adult vaccines… it will be on your mind as well. P8* *Even we didn’t think that adults should be vaccinated… after the pandemic, everyone got interested, even the youth and those 40–50 years old. I think their stances have changed. P14* *An array of new vaccines was released after the COVID-19 ones. Adult vaccination became ‘cool’ in a sense. P19*
Strengthening the value of prevention	*[Trust in vaccines] aided our patients’ trust in other preventive measures and services as well. P6* *Everyone loved prevention, as they witnessed the importance of mass vaccination in practice. P15*

**Table 3:** Weakened vaccine confidence after the COVID-19 pandemic described by main themes and illustrative quotes.

Main Theme	Quote
Complacency *(especially in regions not hit hard by the pandemic)*	*We didn’t experience tragic moments during the pandemic in our area. We had 3–4 deaths in the difficult phases, and everything went smoothly afterwards. We did not experience what Italy went through… we just saw it on the TV and people weren’t that concerned. P2* *Once the SARS-CoV-2 virus was mild, the public was reassured, and they didn’t want to go through vaccines all over again. P17*
Fear *(unprecedented speed of development, reported side effects, need for specialized medical personnel in vaccination centers)*	*There was fear about the vaccine, right? This fear did damage vaccines… When vaccinations are performed in specialized vaccination centers, people think that ‘this has side effects and I have to do it there’… things were extreme and it was eventually against immunizations… people think that something else is going on and you must get vaccinated at specialized centers with doctors present. P5* *There was fear…’yes, but how fast? When other vaccines were studied for 10 years, these ones are out in 6 months. What’s behind all that?’ P12*
Unmet expectations *(contracting and transmitting the virus)*	*People had something different in mind regarding the COVID-19 vaccine… they had different expectations from the vaccine; they thought that they would vaccinate and never contract COVID-19 again. They were thus disappointed. It was a massive [blowout]… and this damaged all vaccines eventually. P4* *There was the belief that vaccines help, avoid contracting the disease and have everything go well. Once they started contracting COVID-19 and vaccinated individuals with no chronic conditions lost their lives, belief in vaccines was lost. P21*
Overexposure	*The frequent presence of academics and non-academic [physicians] on TV, in panels, and in debates regarding immunization did damage vaccines. P4* *Health providers exposed on TV did exhaust citizens. P13*
Compulsory vaccination *(justifiable due to the weak health system and cultural norms, disheartening citizens, increasing resistance)*	*I consider the compulsory nature of vaccination to have been a mistake… you blackmailed individuals in a sense. P16* *[Compulsory vaccinations] did not increase confidence in vaccination, I am sure. P18* *I think that both for the COVID-19 and the other vaccines, adopting compulsory measures managed to increase the public’s resistance. P22*
Confusing information	*COVID-19 vaccination was communicated rather offensively and not right…and within the medical community, there were those who were refusing vaccines. Sometimes the castles fall from within. P12* *Many scientists, so-called experts, said a lot and people got confused… today’s elder are not who they used to be. They know how to roam the web, read, ask and choose according to their criteria their pathways of action. I think people got very confused this way. P25*
Duty and fatigue	*They think that since they vaccinated against COVID-19, they fulfilled their duty and now we are not to ask them for something else. They think that they did their duty and now we should leave them unbothered. P11* *If you tell them about a new vaccine, they are acting strange… ‘another one? How many must I do? Please tell me that they are not like the ones for COVID?’ P16* *‘I have done enough, I have been vaccinated multiple times against COVID-19, I did my duty, I don’t need to get the pneumococcal jab, let me be’. P17*
Reinforcement of former beliefs	*I think that those opposing vaccinations became even more adamant. Hesitancy was prevalent beforehand… I remember that hesitancy began with the H1N1 vaccines in the past… COVID-19 was like a vindication for them. P18* *There are walls whose concrete got even more reinforced after the pandemic. P20*

## Data Availability

The original contributions presented in this study are included in the article. Further inquiries can be directed to the corresponding author.

## Supplementary Materials

The following supporting information can be downloaded at: https://www.mdpi.com/article/10.3390/vaccines14050415/s1. File S1: The part of the interview guide relevant to the findings of this article; File S2: Example of coding and theme development process; File S3: Standards for Reporting Qualitative Research (SRQR) [19].
