# Supplementary material for: Building on Vaccine Confidence in the Aftermath of the Pandemic: A Qualitative Study in Primary Care Physicians
Source: Vaccines (Basel). 2026 May 4;14(5):415. doi: 10.3390/vaccines14050415 (PMC13211669; doi:10.3390/vaccines14050415)
Supplement: Supplementary file 1 [file vaccines-14-00415-s001.zip › Supplementary file 2.pdf]

**S2.** Example of coding and theme development process.

| Raw Data (Participant Quotes) | Identified Codes | Sub-theme | Main Theme | Overarching Theme |
|---|---|---|---|---|
| *An awareness campaign from official national actors... they are more valid institutions… the Health Ministry, the National Public Health Organization and academics can produce campaigns that revolve around information about the disease, the importance of vaccination, how one can get vaccinated. P9* | - Awareness campaign<br>- National stakeholders<br>- Trust<br>- Disease education<br>- Value of vaccination<br>- Process of vaccination | - Content of awareness campaigns<br>- Actors designing and delivering awareness campaigns | Awareness campaigns | Strengthening the general population's health literacy |
| *There should be a record… we should be organized… we should know, beyond any doubt, what has been done, when and by whom. Right now, we are counting on everyone's memory and honesty. We have to believe whatever they say. P5* | - Vaccination registry<br>- Establishment<br>- Vaccination history<br>- Uncertainty of current status | - Unreliable/ Unretrievable information on vaccination history at the moment<br>- Creation of vaccination registry | Establishing an adult vaccination registry | Implementing health policies promoting immunization |
| *It is simpler to get vaccinated in a pharmacy. The shot gets demystified and people are not scared. The level of trust [in vaccines] is elevated as the health provider in your neighborhood, the one who takes good care of you, is the one performing the vaccination. P5* | - Vaccination location<br>- Pharmacy<br>- Less fear<br>- Trust in vaccines<br>- Trust in Health providers<br>- Familiar environment | - Offering vaccination services in pharmacies<br>- Strengthening trust and perception of safety in immunization | Vaccination sites | Implementing health policies promoting immunization |
