# Supplementary material for: Building on Vaccine Confidence in the Aftermath of the Pandemic: A Qualitative Study in Primary Care Physicians
Source: Vaccines (Basel). 2026 May 4;14(5):415. doi: 10.3390/vaccines14050415 (PMC13211669; doi:10.3390/vaccines14050415)
Supplement: Supplementary file 1 [file vaccines-14-00415-s001.zip › Supplementary file 1.pdf]

S1. The part of the interview guide relevant to the findings of this article

- The COVID-19 pandemic and adult immunization:

    (a) How, if any, did patients' needs shift in the aftermath of the pandemic?

    (b) Have you noticed a shift in patients' stances and beliefs towards adult immunization?

    (c) Have you noticed a shift in patients' practices towards adult immunization?

- How can we best increase the general population's confidence in vaccines and adult vaccination?
